# Characterization of Fatty Acids, Polysaccharides, Amino Acids, and Minerals in Marine Macroalga *Chaetomorpha crassa* and Evaluation of Their Potentials in Skin Cosmetics

**DOI:** 10.3390/molecules26247515

**Published:** 2021-12-11

**Authors:** Haresh S. Kalasariya, Nikunj B. Patel, Akanksha Yadav, Kahkashan Perveen, Virendra Kumar Yadav, Faris M. Munshi, Krishna Kumar Yadav, Shamshad Alam, You-Kyung Jung, Byong-Hun Jeon

**Affiliations:** 1 Microbiology Department, Sankalchand Patel University, Visnagar 384315, India; hskalasariya.fsh@spu.ac.in (H.S.K.); nbpatel.fsh@spu.ac.in (N.B.P.); 2 Department of Home Science, Institute of Science, MMV, Banaras Hindu University, Varanasi 221005, India; akankshayadav594@gmail.com; 3 Department of Botany & Microbiology, College of Science, King Saud University, Riyadh 11495, Saudi Arabia; kperveen@ksu.edu.sa; 4 School of Sciences, P P Savani University, NH 8, GETCO, Near Biltech, Village, Dhamdod, Kosamba 394125, India; Virendra.yadav@ppsu.ac.in; 5 Department of Civil Engineering, College of Engineering, King Saud University, P.O. Box 800, Riyadh 11421, Saudi Arabia; fmunshi@ksu.edu.sa (F.M.M.); salam@ksu.edu.sa (S.A.); 6 Faculty of Science and Technology, Madhyanchal Professional University, Ratibad, Bhopal 462044, India; envirokrishna@gmail.com; 7 Department of Chemistry, Yonsei University, Wonju 26493, Korea; fts135@yonsei.ac.kr; 8 Department of Earth Resources and Environmental Engineering, Hanyang University, Seoul 04763, Korea

**Keywords:** marine algae, *Chaetomorpha crassa*, HRLCMS-QTOF, seaweed, cosmetic

## Abstract

Cosmetic industries are highly committed to finding natural sources of functional active constituents preferable to safer materials to meet consumers’ demands. Marine macroalgae have diversified bioactive constituents and possess potential benefits in beauty care products. Hence, the present study was carried out to characterize the biochemical profile of marine macroalga *Chaetomorpha crassa* by using different techniques for revealing its cosmetic potentials. In results, the FTIR study characterized the presence of different bioactive functional groups that are responsible for many skin-beneficial compounds whereas six and fifteen different important phycocompounds were found in GCMS analysis of ethanolic and methanolic extracts, respectively. In the saccharide profile of *C. crassa*, a total of eight different carbohydrate derivatives were determined by the HRLCMS Q-TOF technique, which showed wide varieties of cosmetic interest. In ICP AES analysis, Si was found to be highest whereas Cu was found to be lowest among other elements. A total of twenty-one amino acids were measured by the HRLCMS-QTOF technique, which revealed the highest amount of the amino acid, Aspartic acid (1207.45 nmol/mL) and tyrosine (106.77 nmol/mL) was found to be the lowest in amount among other amino acids. Their cosmetic potentials have been studied based on previous research studies. The incorporation of seaweed-based bioactive components in cosmetics has been extensively growing due to its skin health-promoting effects.

## 1. Introduction

Cosmetic products are a topical combination of cosmetic and pharmaceutical in cosmeceutical with biologically active ingredients to have medicinal or drug-like benefits to improve skin health [1]. Due to the modern lifestyle and increasing beauty concerns, the beauty care industries are growing each year all over the world. To meet the demand of consumers, these industries are moving towards the unstoppable use of synthetic cosmetics and ingredients. Due to the ineffectiveness of synthetic ingredients, they may accumulate in skin layers and create toxicities and harm to normal skin health. According to Kerdudo et al. [2], hydroxybenzoic acid esters (parabens), which are widely used in cosmetic formulations, were reported as harmful to the skin as well as causing an increased incidence of breast cancer and malignant melanoma. Phthalates, as an example, are commonly found in several cosmetic products that can cause DNA modifications and damage as proved in human sperm cells [3]. Some of these chemicals can cause harmful effects in animal studies such as decreased sperm counts, altered pregnancy outcomes, male genitalia congenital disabilities, etc. [4]. As a result, people have changed their preferences and opted for natural cosmetic products. Hence, the ever-expanding market for skincare products and continual search for an alternative natural ingredient has led to the development of a multitude of skin cosmetic formulations [5].

Seaweeds, also known as macroalgae are eukaryotic, multicellular, macroscopic, marine photosynthetic eukaryotic organisms that are ubiquitously distributed along with all kinds of coasts from tropical regions to polar regions. It is normally inhabiting the intertidal and sub-tidal regions of coastal areas [6]. Macroalgae are mainly classified into three major types, namely Brown algae (Phaeophyta phylum, Phaeophyceae class), red algae (Rhodophyta phylum, Rhodophyceae class), and green algae (Chlorophyta phylum, Chlorophyceae class). In which, green and red algae belong to the Plantae kingdom whereas brown algae belong to the Chromista kingdom [7]. Marine macroalgae are a rich source of structurally diverse biological active constituents. Algae contain ten times greater diversified phycocompounds than terrestrial plants. The biologically active constituents found in marine macroalgae have multiple activities, which allow them to be used as an active constituent in formulations. The utilization of macroalgae in cosmetic applications is based on their valuable bioactive compounds, such as carbohydrates, proteins, amino acids, lipids, fatty acids, phenolic compounds, pigments, vitamins, and minerals [8] Broad applications of marine macroalgae are based on valuable bioactive compounds and potential bioactivities. For the development of cosmetic formulations, seaweed-derived compounds have been given considerable importance.

*C. crassa* is a hair-shaped green alga, similarly known as spaghetti algae. It is rapid-growing, hardy, and possesses a bright green to greenish-white appearance. It mainly absorbs nitrate and phosphate for its growth. The thalli of this algae constitute unbranched filaments forming clumps resembling a ball of fishing line. Filaments of *C. crassa* consist of cylindrical cells about 600 to 700 µ in diameter. It is very commonly found in various sea sites on the Western Coast of Gujarat, India. It has maximum growth in an intertidal (supralittoral) zone of the beyt Dwarka sea coast due to the availability of moderate temperature in the growing season from November to February. There is no previous reporting on an application of *C. crassa* in any cosmetic formulation or application. This alga can make a new benchmark in the cosmetic sector after successful experimentation and clinical studies. Besides, this study will be helpful to know the cosmetic potential of *C. crassa* and become helpful to lead this alga from sea to beauty market after its biochemical profiling.

Many previous research studies demonstrated the wide range of biological activities of seaweed-derived active molecules that can offer a variety of skin benefits. The present study aims to characterize green alga *Chaetomorpha crassa* derived bioactive compounds, polysaccharides, fatty acids, amino acids, minerals by using different characterization techniques and to reveal its cosmetic potentials by previous research studies.

## 2. Materials and Methods

### 2.1. Collection of Chaetomorpha crassa

**Figure 1:**
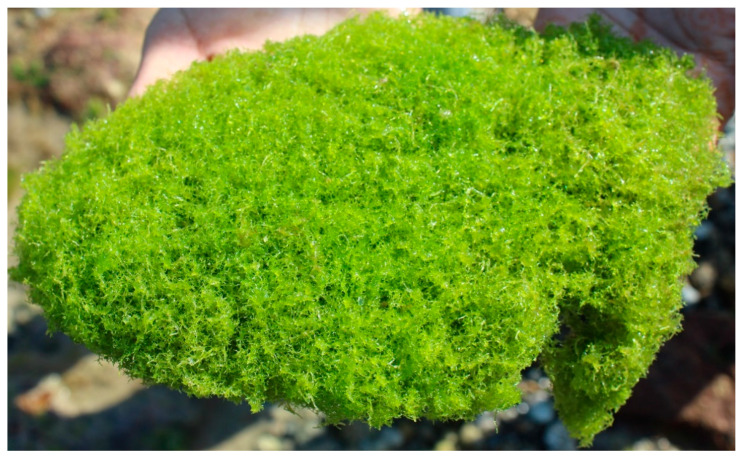
*Chaetomorpha crassa* (Chlorophyta): isolated sample of the macroalga (from Beyt dwarka sea coast, 22°28′43.5″ N 69°08′08.8″ E).

**Figure 2:**
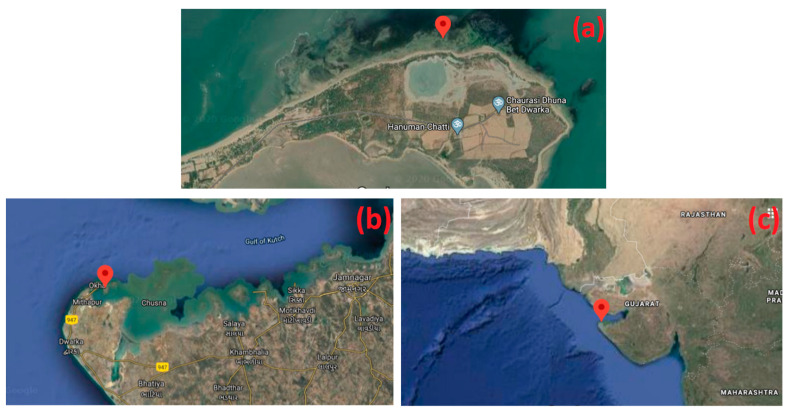
Geographical views of sample site: ((**a**)–Site view) & ((**b**)–state view) Location of sampling site at 22°28′43.5″ N 69°08′08.8″ E (**c**) Location of sampling site in respect to the state of Gujarat, India.

The fresh algal sample was collected in polythene bags containing seawater from the coastal line of Beyt Dwarka (22°28′43.5″ N 69°08′08.8″ E), Western coast of Gujarat, India, and transported (10 °C) to the laboratory. Sample collection sites were illustrated in Figure 1. The identification of the collected sample was carried out with the help of Dr. Nilesh H. Joshi, at the Fisheries Department, Junagadh Agriculture University, Okha. Identified image of *C. crassa* is revealed in Figure 2. The sample was collected by handpicking from the intertidal zone during low tide conditions (Precipitation: 0%, Humidity: 55%, Wind: 11 km/h, 27 °C, February 2021). Then the algal material was washed thoroughly to remove extra epiphytes, salts, and marine debris in running tap water. Finally, it was washed with distilled water and allowed to shed dry (room temperature) for five to seven days, until the moisture was completely removed. The dried material was ground to a fine powder using an electric grinder and stored in air-tight containers at −20 °C temperature until further analysis.

### 2.2. Characterization of Phycocompounds by GCMS Analysis

#### 2.2.1. Extract Preparation

The extraction was carried out by using 500 g of dry powder in ethanol (80%) at 70 °C by continuous hot percolation using the Soxhlet apparatus. It was continued for 24 h, and the ethanolic extract was then filtered and kept in a hot air oven (RDHO 80, Dry hot air oven, REMI, Bengaluru, India) at 40 °C for 24 h to evaporate the ethanol (Sigma-Aldrich, Bengaluru, India) from it. The extract obtained was concentrated to dryness under decreased pressure (150 mbar) at 20 °C using a rotary vacuum evaporator (Sigma Scientific, Nanganallur, Chennai, India). The obtained residue was kept separately in airtight containers and stored in a deep freezer (−20 °C, Esquire Biotech, Chennai, India).

#### 2.2.2. GCMS Characterization

For GC-MS analysis, the sample was injected into an EB-5 column in the JMS-T100GCV GC model used for chromatographic separation. Helium was used as carrier gas with a flow rate of 1 mL/min; the injector was operated at 200 °C and column oven temperature was programmed as 50–250 °C at a rate of 10 °C/min injection mode. The following MS conditions were used: For mass spectrometry analysis, AccuTof Mass from jeol was used. ionization voltage of 70 eV; ion source temperature of 250 °C; interface temperature of 250 °C; the mass range of 50–600 mass units. A gas chromatogram was obtained and the mass spectrum of the unknown phycocompounds was compared with the spectrum of the known components stored in the NIST library version 2005.

### 2.3. Fatty Acids Characterization by GCMS Analysis

The prepared fine powder of algal material was extracted with methanol (anhydrous, 99.8%, Sigma-Aldrich, Bengaluru, India) in the ratio of 1:10 (*w*/*v*) in a flask for 72 h. The mixture was filtered in a separate container. This process was repeated two times with the same residues using a fresh solvent. After the collection of supernatants, the excess solvent was removed by a rotary evaporator (Sigma Scientific, Nangainallur, Chennai, India) and followed for further analysis. The GC–MS analysis was carried out using a JMS-T100GCV gas chromatograph used and coupled to a mass detector AccuTof Mass from jeol. For analysis, the sample was injected into an HP-5 column. The specifications include ion source temperature of 250 °C; interface temperature of 250 °C; mass range of 50–600 mass units and helium flow rate as one mL/min. The ionization voltage was 70 eV. The samples were injected in split mode as 1:10. A gas chromatogram was obtained and the mass spectrum of each unknown compound was interpreted with the spectrum of the known compounds stored in the NIST (National Institute Standard and Technology) library version 2005. The name, molecular weight, and structure of unknown compounds were retrieved. The relative percentage amounts of each compound were calculated by comparing its average peak area to the total area.

### 2.4. Characterization of Functional Groups by FTIR Study

FTIR is the most powerful tool for identifying the types of functional groups present in compounds. The wavelength of light absorbed is characteristic of the chemical bond as can be seen in the spectrum. In analysis, 3000 Hyperion Microscope with Vertex 80 FTIR System, Bruker, Germany. A total of 5 mg of dried powder was taken, KBr (Potassium bromide) (FT-IR grade, ≥99% trace metal basis, Sigma-Aldrich, India) was added and evenly mixed with the sample to obtain a homogenized fine powder. The prepared powder was then placed in the molds and pressed using mechanical support for half a minute using a sterile spatula. The prepared pellet of the sample with the molds was shifted on the pan and was proceeded for analysis. The scanning was performed at wavelength 400–4000 cm^−1^, The scanning results were displayed in % transmission analysis. The functional groups of the components were separated based on their peak ratio (based on the peak values in the region of IR radiation).

### 2.5. Elemental Analysis by ICP AES

#### 2.5.1. Chemical Preparation

Standard pure grade reagents (Merck, Germany) were used for the above digestion procedure throughout the experiment. Other solvents such as nitric acid, hydrochloric acid, hydrogen peroxide, and hydrogen fluoride were of analytical reagent grade (Merck). All glassware as well as plastic wares were soaked with 10% nitric acid (ACS reagent, Sigma-Aldrich, India) overnight and then rinsed with distilled water before use.

#### 2.5.2. Microwave-Assisted Digestion and Detection

Approximately 0.05 g of sample was weighed directly into the TFM-PTFE (second-generation modified Polytetra-fluoro-ethylene-) vessels, to which the reagents were added (3 mL Hydrochloric acid + 1 mL Nitric acid + 1 mL Hydrogen fluoride + 1 mL Hydrogen peroxide) (Sigma-Aldrich, India) and the vessels were closed immediately. The operational conditions and the heating program were carried out after subjecting the above mixture to a microwave digestor (Titan Microwave system, PerkinElmer, Thane, India) according to the conditions: 130 degrees ramp time 15 min hold time 10 min, and 190 degrees ramp time 15 min hold time 10 min. After cooling these vessels at 70 °C, they were vented and opened. Then, made up to 25 mL by adding Milli-Q water into the vessels and then shaken thoroughly to dilute any possible rest of the colloids attached to the vessels’ walls. Blank was also subjected to microwave digestion. The Inductively Coupled Plasma Atomic Emission Spectrometer (ICP-AES) instrument model used was ARCOS, Simultaneous ICP Spectrometer from SPECTRO Analytical Instruments GmbH, Germany. The software used was Smart Analyzer Vision 5.01.0921. The detector was a charge-coupled device (CCD). All the samples were analyzed in triplicate.

### 2.6. Determination of Total Amino Acid Profile by HRLCMS-QTOF

#### Acid Hydrolysis Procedure

100 mg sample was weighed into a test tube. Add 12 mL of 6 N HCl in it and the tubes were heat sealed after filling pure nitrogen gas. Keep the test tube in Hot air oven (RDHO 80, Dry hot air oven, REMI, India) at 120 °C temperature for 16 h for hydrolysis. After hydrolysis, the content was removed and filtered. Flash Evaporation was carried out to remove traces of HCl. The residue was made up to a definite volume with 0.05 N HCl. It was filtered again through a Whatman membrane filter of 0.45-micron size. The sample was injected into 6550 iFunnel Q-TOFs, Agilent Technologies, USA. The column used is Poroshell HPH C18-4.6 × 100 mm, 2.7-micron. The oven temperature was maintained at 60 °C and analysis was carried out with the non-switching flow method and fluorescence detection after post-column derivatization. The Standard of amino acids was also run to calculate the concentration of the amino acid depending on the standard chromatogram.

### 2.7. Carbohydrate Profiling

#### 2.7.1. Acid Hydrolysis

The sample was first dried in an oven at 40 °C for 48 h, then powdered to obtain approximately uniform fine particles. A total of 100 mg of the sample was placed in screw-cap tubes (16 mm 25 mm) and add 10 mL of 2 M hydrochloric acid containing 1% phenol. Then, the tubes were closed under nitrogen gas and kept in an electric oven (REMI, Maharashtra, India) at 80 °C for 3 h, allowing it to cool, and their contents were vacuum-filtered through Whatman no. 41 paper. After filtration, the filtrate was diluted to 25 mL with ultrapure water in a volumetric flask and the resulting liquid was again membrane-filtered to get the hydrolysate.

#### 2.7.2. HRLCMS Q-TOF Analysis

Polysaccharides present in the extract of *Chaetomorpha crassa* were identified using an advanced analytical technique, HRLCMS QTOF. The test sample was analyzed by HRLCMS Q-TOF analysis (6550 iFunnel Q-TOFs, Agilent Technologies, USA) and characterized by setting its scanning range from 150–1000 m/z for MS/MS Dual AJS ESI in the mode of ionization. The Hypersil GOLD C18 Column, size 100 mm × 2.1 mm × 3 μm was used. Resolved peaks were further identified with the help of reported values from the literature. Above analysis was performed with the following settings: Source parameters include: Gas Temp: 250 °C; Gas Flow: 3 L/min; Nebulizer: 35 psig; Sheath Gas Temp: 300; Sheath Gas Flow: 11; Scan source parameters: VCap: 3500; Nozzle Voltage: 1000 V; Fragmentor: 175; Skimmer1: 65; OctopoleRFPeak: 750; Auxillary parameters: Draw Position Offset: 0.0 mm; Eject Speed: 100.0 μL/min; Draw Speed: 100.0 μL/min; Vial/Well bottom sensing: Yes; Sample Flush Out Factor: 5.0; Wait Time After Drawing: 2.0 s.

## 3. Results

### 3.1. Phycocompounds Characterization by GC-MS Analysis

**Figure 3:**
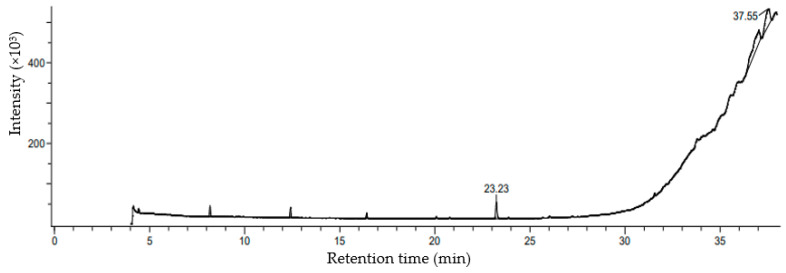
The chromatogram obtained by GC/MS analysis of the ethanolic extract of *C. crassa*. Comment: Split1:10;80-1M-6-200-2M-8-275-5M-5-280-EB5. Instrument configuration: JMS-T100GCV. JEOL The AccuTOF GCv: 40.00.650.00.

The GCMS analysis of ethanolic extract showed six compounds that were identified based on retention time, % peak area, molecular formula, and molecular weight. A gas chromatogram with different peaks is represented in Figure 3. A total of six different compounds were detected from the ethanolic extract of *Chaetomorpha crassa* (Table 1).

Among the identified compounds, Dodecanoic acid,1,2,3-propanetriyl ester is found to be the major compound which revealed the largest peak (48.89%) with the retention time (37.03 min). Chemical structures of obtained compounds are depicted in supplementary data Table S1.

### 3.2. Fatty Derivatives Characterization by GC-MS Analysis

**Figure 4:**
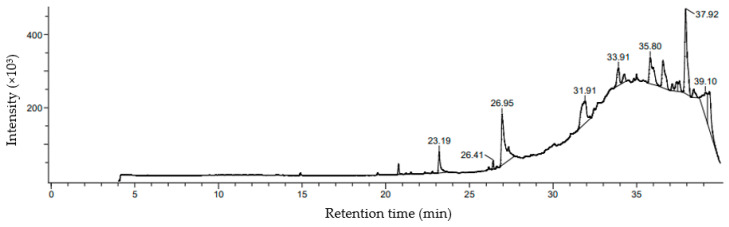
The chromatogram obtained by GC/MS analysis of the methanolic extract of *C. crassa*. Comment: Split1:10;80-1M-6-200-3M-8-275-5M-5-280-. Instrument configuration: JMS-T100GCV. JEOL The AccuTOF GCv: 35.00.650.00.

In GC-MS characterization analysis, identification of phycocompounds was carried out based on retention time, peak area, molecular formula, and molecular weight. A gas chromatogram with different peaks is represented in Figure 4. Total fifteen different fatty acids were detected from the methanolic extract of *Chaetomorpha crassa* (Table 2).

Among the identified compounds, 9-Octadecenoic acid [Z]-, phenylmethyl ester is found to be the major compound that attained the largest peak (16.64%) with a retention time (39.1 min) whereas Oxirane, tetradecyl- showed the lowest peak area of 0.85% with a retention time of 26.41 min.

E-11-Tetradecenol, trimethylsilyl ether; n-Hexadecanoic acid; Oleic Acid are long-chain fatty acids that belong to superclass lipid and lipid-like molecules. Other lipid-like molecules, 9,12-Octadecadienoic acid [Z]-, phenylmethyl ester; and 6,9,12-Octadecatrienoic acid, phenylmethyl ester, [Z, Z, Z]- were found to be Lineolic acids and derivatives whereas Ergost-5-en-3-ol, acetate, [3β,24R]-, similarly known as Campesterol acetate, was an Ergosterol derivative. Stigmastan-3,5-diene and Stigmastan-6,22-dien, 3,5-dedihydro- were found to be Stigmastane derivatives (steroid derivatives) belonging to the superclass Lipids and lipid-like molecules. Other Lipid-like molecules, 6-Octadecenoic acid, methyl ester, [Z]-; 9-Octadecenoic acid [Z]-, phenylmethyl ester; and Z-8-Methyl-9-tetradecenoic acid were found to be Fatty acid methyl esters (with Fatty Acyls) In addition, Hydroxy secosteroid compound 9,10-Secocholesta-5,7,10[19]-triene-3,24,25-triol, [3β,5Z,7E]-, similarly known as calcidiol (Lipid-like molecule), was found. Moreover, Oxirane, tetradecyl- is Epoxides belongs to Organ heterocyclic compounds whereas 5-Methyl-Z-5-docosene was Unsaturated aliphatic hydrocarbons belongs to superclass Hydrocarbons. Lastly, hydrocarbon derivative or aldehyde derivative Tridecanedial was found which is applicable for the perfuming agent but still not clear.

### 3.3. Fourier Transform Infrared Spectrophotometer (FTIR) Analysis

**Figure 5:**
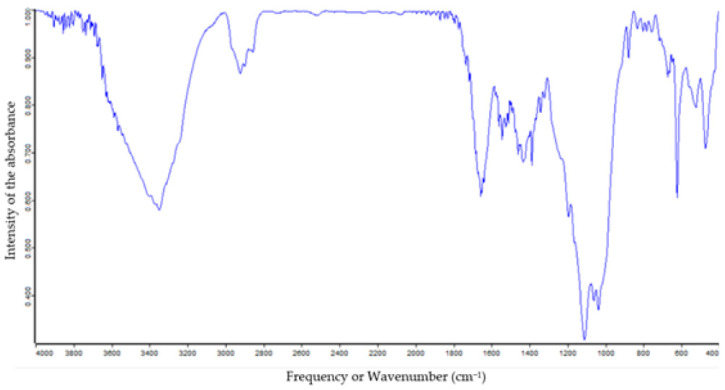
FTIR spectrum of *C. crassa* powder by KBr pellet method. (Pick values tabulated in Table 3).

FTIR spectrum analysis was used to characterize the functional group of active constituents based on the peak value in the infrared region. The FTIR spectrum is illustrated in Figure 5. The result of FTIR peak values and functional groups is represented in Table 3. The more intense band occurring at 3648 cm^−1^, 3628 cm^−1^, 3566 cm^−1^, 3346 cm^−1^, 2925 cm^−1^, 2859 cm^−1^, 1888 cm^−1^, 1869 cm^−1^, 1714 cm^−1^, 1660 cm^−1^, 1560 cm^−1^, 1540 cm^−1^, 1517 cm^−1^, 1432 cm^−1^, 1192 cm^−1^, 1111 cm^−1^, 1041 cm^−1^, 875 cm^−1^, and 674 cm^−1^ corresponding to O-H/N-H/C-H/C=0/C=N/C-N/N-O/CO-O-CO/C-Cl/C-Br/C=C. This result of FTIR spectroscopic analysis revealed the presence of amine, alcohol, phenol, aromatics, alkanes, alkenes, anhydride, carboxylic acids, ester, sulfoxide, nitro, ether, and substituted constituents in the extract of *Chaetomorpha crassa*.

### 3.4. Determination of Elements by ICP-AES Analysis

Minerals are metallic elements present in various forms in diversified marine algae. They are highly applicable in skin cosmetics formulations. The macromolecules such as Silicon, Potassium, Calcium, Iron, and Magnesium are among the minerals which are present in significant amounts in selected marine alga *C. crassa* among screened elements. The result showed that Silicon was the highest in amount whereas Copper is the lowest in amount. The percentage of minerals follow Si > K > Ca > Fe > Mg > Na > Zn > B > Cu order. Concentrations for each element (in %) are given in Table 4.

### 3.5. Determination of Amino Acids

**Figure 6:**
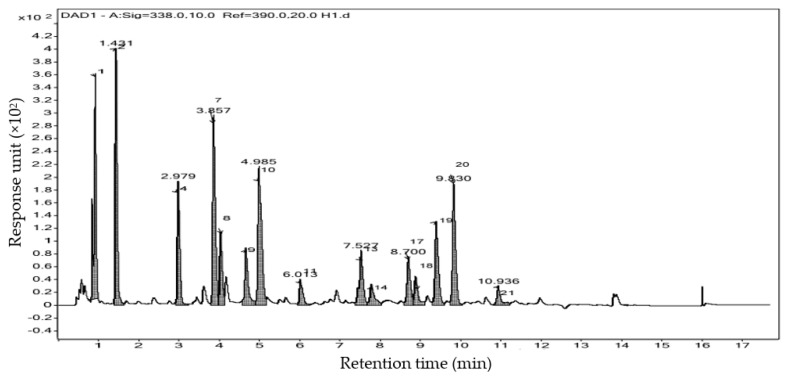
Chromatogram for characterization of different amino acids from *C. crassa* by HRLCMS-QTOF.

Total 21 different amino acids were analyzed in *C. crassa* by HRLCMS-QTOF analysis. A gas chromatogram with different peaks for different amino acids is represented in Figure 6. Among all, Aspartic acid, Glutamic acid, Hydroxyproline, Glycine, and Alanine were found dominant in the detection above 0.5 g/kg. Aspartic acid amino acid (1.6 g/kg) was found to be the highest in amount whereas amino acid Tyrosine (0.2 g/kg) remained lowest in the above measurement. Detected amino acids were following below order in content (nmol/mL): Aspartic acid > Glutamic acid > Hydroxyproline > Glycine > Alanine > Serine > Leucine > Valine > Threonine > Lysine > Phenylalanine > Arginine > Isoleucine > Methionine > Tyrosine. Concentrations for each amino acid (in g/kg) are expressed in Table 5.

### 3.6. Carbohydrate Derivatives Analysis by HRLCMS-QTOF Study

**Figure 7:**
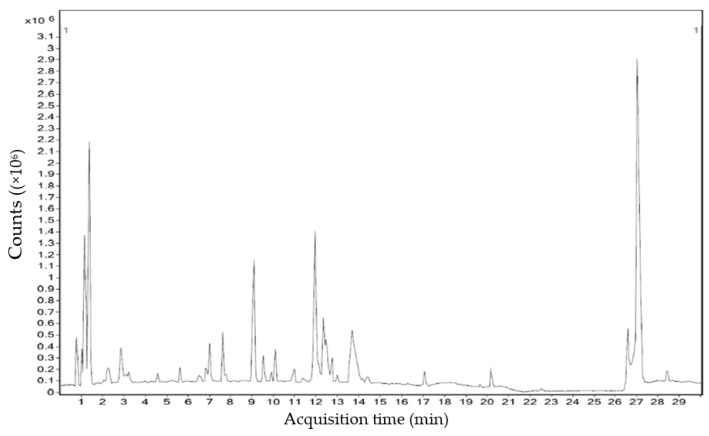
Liquid Chromatogram for polysaccharides’ characterization of *C. crassa* by HRLCMS-QTOF. X-axis: Acquisition time (min); Y-axis: Counts (×10^6^).

HR-LCMS Q-TOF analysis of methanol extract of *C. crassa* showed a total of eight different compounds at different retention times. For this analysis, a liquid chromatogram with different peaks is represented in Figure 7. The obtained seven different carbohydrate derivatives and their details were illustrated in Table 6. By comparison of High-Resolution Liquid Chromatography and Mass Spectra of compounds with the main library of all these constituents were identified and characterized. The chemical structure of each carbohydrate derivative is depicted in Supplementary Table S3. Moreover, HRLCMS Q-TOF analysis showed the presence of some fatty derivatives such as beta-Butoxyethyl nicotinate (RT: 4.356 min); Dihydrocapsaicin (RT: 7.648 min); Manumycin A (RT: 9.103 min); 16-methyl-6Z,9Z,12Zheptadecatrienoic acid (RT: 12.749 min); Axisothiocyanate 3 (RT: 1.465 min); N-(1-Oxooctyl)glycine (RT: 4.03 min) and Phytosphingosine (RT: 10.013 min). Likewise, two peptides, Tyr Val Phe and Tyr Lys Lys were found with different retention times of 3.896 min and 5.175 min, respectively (For chemical structures, see Supplementary Table S4).

In addition, carbocyclic compound, Tiocarbazil; Polycyclic aromatic hydrocarbon, Benzo[a]fluorene; Monocarboxylic acid amide, 4-Hydroxy-3-nitrosobenzamide; and Carbobicyclic compound, (S)-Indenestrol A were reported with different retention times and in different abundance. Benzylisoquinolines (alkaloids), Isocorydine (+) and Vinca alkaloids, 3-α(S)-Strictosidine were found with retention time 4.6 min and 5.642 min, respectively. Organooxygen compound, Metaxalone; Carboxylic ester compound, Scutellarioside II; Acyclic heterone compound, Triphenylphosphine oxide; Aromatic amine compound, Pirimicarb; Paraben, 3,5-Dichloro-4-hydroxy-2methoxy-6-methylbenzoic acid; Dialkylsulfides compound, Dicyclohexyl disulfide (FEMA 3448); Organic amino compound, and Tetradecylamine were obtained with different hits, RT and abundance. Likely, an azamacrocycle and Lactum compound, 34a-Deoxy-rifamycin W; Carbonyl compounds, (+/−)-3-[(2-methyl-3furyl)thio]-2-butanone; Quinoxaline derivative, Chinomethionat; Gamma-amino acid, Gabapentin; Benzamide, Azoxy-1-procarbazine; and 2,2,6,6-Tetramethyl-4piperidinone were presented with different hits.

## 4. Discussion

Marine macroalgae-derived ingredients have been used in skincare applications due to their potential skin benefits. The European Chemicals Agency reported the role of Hydroperoxide, 1-methylhexyl in cosmetic and personal care products for antimicrobial and antioxidant activity [9]. It is also applicable as dyestuffs during oxidative hair dyeing, Dicarboxylic acids and derivatives, Oxalic acid, isobutyl nonyl ester have been demonstrated as having cosmetic benefits as a pH adjuster, fragrance enhancer, as well as skin conditioning agent-emollient [10]. Dodecanoic acid,1,2,3-propanetriyl ester, also known as Trilaurin, presented its use in skin conditioning agents (for maintaining skin functions) as well as occlusive or viscosity increasing agents [11]. Rajamanikyam et al. [12] studied the antibacterial activity of phthalic acid ester against different bacterial species such as *S. epidermidis*, *S. aureus*, *P. aeruginosa*, *Klebsiella pneumoniae*, and *Bacillus subtilis*. In addition, Bakkali et al. [13] suggested the use of phthalic acid ester compound in skin cosmetic applications. Patil et al. [14] suggested the use of alkane class compound Tritriacontane, 13-decyl-13-heptyl- for skin benefits as emollients. Two markedly available products Jarcane™ 16 and Jeechem^®^ NDA-H reported containing Hexadecane. The former product is useful for waxes, moisturizing agents, softening texturing agents, and solubilizers whereas the second product is used for softening texturing agents, thickeners stabilizers, and emollients.

Fatty acids have an important role in the maintenance of skin functions. First, fatty acids are an important player in normal skin barrier functions. Long-chain fatty acids become helpful in moisture retention, prevent the entry of harmful substances. They also maintain certain metabolic processes of skin cells. They can provide moisturizing and anti-inflammatory effects. Likewise, medium-chain fatty acids protect against inflammation and tumor factors. In addition, short-chain fatty acids can provide anti-inflammatory effects and initiate an immune response [15]. Cui et al. [16] suggested the antibacterial and anti-inflammatory properties of fatty acids. In addition, fatty acids have some other skin benefits in moisturization and lubrication of the skin [17].

A previous research study reported the presence of various biologically active constituents such as alkanes, aldehydes, alcohols, ethers, ketones, amides, and carboxylic acids by FTIR spectroscopic study [18]. FTIR has been revealed to be a significant means for the classification and differentiation of intimately relevant species of diverse organisms [19]. Janakiraman et al. [20] showed alkyl halides and alkanes function as groups responsible for antimicrobial activity against *Staphylococcus aureus*, *Candida albicans*, and *Escherichia coli*. The presence of functional groups such as alcohol, aromatic, amine, and carboxylic acids are responsible for the antibacterial activity, which was characterized by FTIR analysis [21]. Likewise, Ambedkar et al. [22] revealed carboxylic acid, alkene, aldehyde, unsaturated ketone, phenol, and halogen groups by FTIR study in ethanolic extract and their antibacterial activity against *S. aureus*, *Bacillus subtilis*, *E. coli*. Alcohol group-containing compounds such as triclosan, chlorobutanol, chlorocresol, phenoxyethanol, dichlorobenzyl alcohol, benzyl alcohol, chlorophene, etc. are useful as preservatives [23]. The European Commission (EC) reported the use of the alcoholic compound triclosan in cosmetic applications mouthwashes, toothpaste, hand soaps, face powder, and body soap [24]. Moreover, the aldehyde group-containing compounds such as formaldehyde are a preservative in shampoos, shower gel, and liquid soaps [25]. Culler et al. [26] found a relationship between alkyl, amide, amines-containing compounds, and antimicrobial activity. In addition, nitrogenous compounds present antibacterial and antifungal activities by a different mode of action [27]. Regarding another nitrogen compound, triclocarban inhibits the growth of Gram-positive bacteria include Methicillin-resistant *Staphylococcus aureus* and resistant *Enterococcus* sp. [28]. Another nitrogen compound, piroctone olamine, reduces microbial colonization by inhibiting the growth of *Malassezia* spp. by showing antifungal activity [29]. e-CFR [30] reported the role of alcoholic and halogenated compounds in skincare products for cleaning and bacterial reduction application as antibacterial soap bars, antibacterial wipes, and disinfectants. Siemer [31], suggested the use of the halogenated compound in face care products for antiseptic cuticle treatment and antiacne as well as showed its effect against *Staphylococcus aureus*, *S. epidermis*, and *Propionibacterium acnes*. Some alcohol and halogen group-containing compounds have useful applications in deodorants and antiperspirants by inhibiting the growth of Staphylococci and Diphtheroids of the Corynebacteriaceae family [32].

Many previous research studies confirmed the beneficial role of minerals for skin health benefits. Elias et al. [33] reported the role of calcium in hemostasis as well as a key regulator of epithelialization, which is critically important for the differentiation of basal keratinocytes to corneocytes. Ca^++^ acts as a key modulator in the locomotion of keratinocytes and promotes wound healing activity in vivo. Potassium and Sodium both demonstrated cellular and extracellular electrolytes and osmolytes for maintaining cell membrane potential. Concerning the skin benefits, Magnesium is beneficial for skin nourishment with a solution prepared from Dead Sea salts [34]. Denda et al. [35] revealed the effects of topical applications of Magnesium and Calcium salts in skin repair. Topical treatment with 5% MgCl_2_ before UVB radiation reduces the number of Langerhan cells in the epidermis as well as decreases antigen-presenting activity in the skin [36]. Although, Zn appears to be safe and effective in many topical formulations. The FDA of the United States recognizes three zinc compounds, zinc acetate, zinc carbonate, and zinc oxide for safe and effective use as topical skin protectants [37]. Some research studies suggested that the presence of zinc in tissue is important in wound healing activity. The research study demonstrated that zinc oxide can be used in topical sunscreen formulations to some extent due to its safe and effective use for skin rashes as well as eczema, impetigo, ringworm, ulcers, pruritus, and psoriasis [38]. Iron is another important most abundant trace metal with various skin beneficial activities. Mainly, it plays a major role in skin-relevant procollagen-proline dioxygenases [39]. Interestingly, Iron is helpful against UV-induced damage by expressing a higher amount of Iron in UV-exposed skin (53.0 ppm) than unexposed skin (17.8 ppm) [40]. Moreover, Copper plays a role in the enhancement of collagen formation and improves skin health. In beauty products, copper-peptides were proven to relax skin irritation., improve skin elasticity, firmness, repair photodamage, reduce fine lines, and antiwrinkle activity with some other benefits [41].

Previous research studies confirmed that the amino acids demonstrated anti-inflammatory, antioxidant, photoprotection, moisturizing, antiwrinkle, antielastase, and skin health benefits [42]. Histidine, Lysine, and Arginine are the three most common amino acids for skincare products as well as Glycine, Proline, and Leucine, usefully abundant in collagen. Likewise, a combination of Lysine and Arginine can effectively treat wound healing, while the combination of Proline and Leucine can repair skin wrinkles [43]. Veis and Anesey [44], reported very important collagen and elastin functions of Lysine. Supplementation of Lysine has been useful for the prevention of acne and cold sores. Moreover, Choi et al. [45] demonstrated the role of Tripeptide containing Lysine and Histidine as a skin moisturizer in a skincare product. Isoleucine, Leucine, Valine plus Arginine, Glycine, and Proline significantly enhanced the synthesis of dermal collagen in hairless mice against UV radiation [46]. A combination of Leucine with Glycine and Proline was also used for the attenuation of skin wrinkles [47]. Kawashima et al. [47] reported that topical treatment with a Proline derivative showed an improvement of skin elasticity in Crow’s feet lines on the faces of Japanese women. Serine and Alanine amino acids as moisturizing agents can be used in skincare products. It plays a general role in water retention in the stratum corneum. Yamane et al. [48] suggested the role of leucine and isoleucine on collagen synthesis in the skin. Isoleucine has been also used as a complement component in ceramides-based emollient cream for the treatment of facial atopic eczema [49]. Aromatic amino acids such as Phenylalanine and Tyrosine have an important role in melanin synthesis as a precursor. Melanin is the main skin cutaneous protective pigment because it mainly absorbs the harmful sunlight ultraviolet radiation and prevents DNA damage and skin cancers [50]. In addition, Phenylalanine, as well as Tyrosine, play an important role in melanin synthesis (Melanogenesis) [51]. Methionine can also be used in the treatment of acne and inflammatory cutaneous lesions as antioxidant complexes [52]. Threonine and Serine are useful for keeping hydration in the stratum corneum. Arginine is one of the highly recommended amino acids to enhance the wound healing effect of injured skin [53]. Moreover, Glycine and Proline amino acids are not only present in collagens but are also regulators of collagen synthesis [54].

Antibiotic JI-20A demonstrated antibacterial activity in vitro and in vivo against pathogenic bacterium [55]. Gentamycin C1a also reported antibacterial activity by binding with 30S ribosomal unit, negatively impacting protein synthesis [56]. Loquatoside showed significant antioxidant, anti-inflammatory, antiviral, antibacterial, antifungal, and anticancer effects [57]. Feruloyl C1-glucuronide reported its role as an antioxidant compound [58]. Likewise, antiviral activity was reported by Miglustat (alkylated imino analog of D-glucose) in the previous study [59]. Bactobolin was found to be a broad-spectrum antibiotic agent on different bacterial species [60]. Capryloylglycine has many functional benefits such as antibacterial activity, controller of excessive sebum secretion (antiacne), as well as an antiwrinkle agent. It is also applied as a conditioning agent or as a surfactant to protect the skin from water loss [61]. Sačková and Fedoročko, [62] studied anticancer activities such as antiproliferative, cytotoxic, antimetastatic, and proautophagic whereas non-anticancer activities such as antifungal, antibacterial, anti-inflammatory effects, etc. of Manumycin A. 16-methyl-6Z,9Z,12Z heptadecatrienoic acid reported antioxidant activity in vitro which was studied by Yang et al. [63]. Table 7 represents the different bioactive compounds except for polysaccharides in this analysis with their PubChem ID, molecular formula, molecular weight, retention time, abundance, and hits.

Yang et al. [64] found wound healing benefits as well as immune regulation, antioxidant, antiaging activities of Axisothiocyanate 3. Phytosphingosine enhances moisture levels in the human skin barrier through modulation of the FLG (filaggrin) synthesis pathway and suggested the formation of the Natural Moisturizing factor [65]. Antimicrobial, antiinflammation, and antitumor activities of 4-Hydroxy-3-nitrosobenzamide were found, which may be useful for skin cosmetic formulation [66]. Tetradecylamine, similarly known as Myristamine oxide has been demonstrated as a foam stabilizer and hair conditioning agent in shampoos and conditioners [67]. Additionally, 34a-Deoxy-rifamycin W has been known for antibacterial activity against many pathogenic bacteria. Its derivatives are also active against many Gram-positive and Gram-negative cocci, mycobacteria, and chlamydia [68]. (+/−)-3-[(2-methyl-3furyl)thio]-2-butanone was also reported to have benefits as a flavoring agent and provides a cooling sensation [69]. Antifungal, as well as insecticidal activities, were found from the quinoxaline derivative, Chinomethionat, similarly known as Quinomethionate [70]. Another compound beta-Butoxyethyl nicotinate (Nicoboxil) is useful in cosmetics as an antiaging, antiwrinkle agent and helpful in inhibition of photocarcinogenesis. It was also checked for its anti-inflammatory effects against acne, rosacea, and mustard-induced irritation [71].

There is growing interest and potential towards seaweed-derived molecules in cosmetic formulation due to the skin benefits and applications [72]. However, it is well established that the cosmetic industries became more interested in macroalgae going further for extraction of various active constituents and their utility in cosmetic formulations. Marine algae are a source of new value-added active compounds with scientific evidence revealing their benefits for safer use in cosmetics [73]. Marine algae-derived molecules have wide diverse applications in many skin biological benefits such as anti-inflammatory, antimicrobial, anticellulite, antiviral, anticancer, skin whitening, antiaging, antiwrinkle, photoprotection, moisturizer, collagen-boosting activities, etc. [42,74]. Moreover, many cosmetic companies already use marine algae extracts and derived compounds in their formulations [75]. However, the biochemical profile monitoring of macroalgae presents a problem that industries need to overcome. The development of its cultivation and green extraction procedure presents the major key for this thematic, which is being analyzed with promising benefits [76]. However, the exact mechanisms of some compounds have not been fully explored in performing biological functions. Therefore, further studies are essential to understand the exact mechanisms, as well as more clinical trials, in order to improve the quality of cosmetic products which will be helpful to enhance the consumer’s safety.

## 5. Conclusions

The current findings revealed that the Chlorophyta *C. crassa* is a good source of active ingredients with much cosmetic potential. In the findings, some of the long-chain fatty acids, Linoleic acids and derivatives, Ergosterols derivatives, and Stigmastane derivatives were found that have moisturization, skin barrier functions, antibacterial, antifungal, antiinflammation, immunostimulatory benefits to the skin. Likewise, in the HRLCMS-QTOF characterization study, some carbohydrate derivatives such as 2-Methyl-2-butenyl beta-D-glucopyranoside; Antibiotic JI 20A; Feruloyl C1-glucuronide; N-butyl-1-deoxynojirimycin; N-(1-Oxooctyl)glycine and other phycocompounds were screened that possess antioxidant, antiwrinkle, antiacne, moisturizer, antimicrobial, conditioning agent, etc. skincare benefits. Amino acids and minerals are the most powerful, effective, and non-toxic important ingredients for skin protection against UV damage, antioxidant, anti-melanogenesis, skin whitening, antiaging, and collagen synthesis activities. Aspartic acid, Glutamic acid, Hydroxyproline, Glycine, Alanine, and Serine were obtained in larger amounts among other amino acids whereas Si, K, Ca, Fe and Mg elements were found higher in *C. crassa* as compared to other elements. This study revealed a very good biochemical profile and great cosmetic potential of marine alga *C. crassa*. These algae and their derived molecules may be useful in skincare applications after successful experimentation. Moreover, there is growing interest and potential towards seaweed-derived molecules in cosmetic formulations due to their skin benefits and applications. Apart from that, phycocompounds from marine algae are more effective when used in cosmetic formulations as they are less harmful as well as natural for the skin structure compared to synthetic compounds.

By different biochemical characterizations, this alga represented a good number of potential ingredients. *C. crassa* or derived compounds can be used as or as a part of a moisturizer, photoprotection formulation, conditioner, skin whitening products as well as in an anti-wrinkle or soothing cream formulations. Moreover, it provides antimicrobial protection and physical health to the skin due to the presence of potent constituents. Along with its potential large availability, (with no worry about the cultivation of *C. crassa* in the Beyt Dwarka sea coast), it could easily be used in skincare formulations after successful in vitro evaluations and clinical studies.

## Figures and Tables

**Table 1:** Details of phycochemicals identified through GC-MS Analysis of Ethanolic Extract.

Name of the Chemical Compound	PubChem ID	Molecular Formula	Molecular Weight (g/mol)	Retention Time (min)	Kovats Index (iu)	Peak Area %	SMILE Structure
Tritriacontane, 13-decyl-13-heptyl-	545591	C_50_H_102_	703.3	16.42	4907	5.04	CCCCCCCCCCCCCCCCCCCCC(CCCCCCC)(CCCCCCCCCC)CCCCCCCCCCCC
Hexadecane	11006	C_16_H_34_	226.44	12.41	1612	9.82	CCCCCCCCCCCCCCCC
Hydroperoxide, 1-methylhexyl	12981	C_7_H_16_O_2_	132.2	4.43	1013	4.0	CCCCCC(C)OO
Oxalic acid, isobutyl nonyl ester	6420705	C_15_H_28_O_4_	272.38	8.19	1783	10.57	CCCCCCCCCOC(=O)C(=O)OCC(C)C
Dodecanoic acid,1,2,3-propanetriyl ester	10851	C_39_H_74_O_6_	639	37.03	4336	48.89	CCCCCCCCCCCC(=O)OCC(COC(=O)CCCCCCCCCCC)OC(=O)CCCCCCCCCCC
Phthalic acid, 6-ethyl-3-octyl butyl ester	6423866	C_22_H_34_O_4_	362.5	23.23	2505	21.67	CCCCOC(=O)C1=CC=CC=C1C(=O)OC(CC)CCC(CC)CC

(iu: index unit).

**Table 2:** Details of Phycochemicals identified through GC-MS Analysis of Methanolic Extract.

Name of the Chemical Compound	PubChem ID	Molecular Formula	Molecular Weight (g/mol)	Retention Time (min)	Kovats Index (iu)	Peak Area %	SMILE Structure
n-Hexadecanoic acid	985	C_16_H_32_O_2_	256.42	23.19	1968	2.98	CCCCCCCCCCCCCCCC(=O)O
Oxirane, tetradecyl-	23741	C_16_H_32_O	240.42	26.41	1702	0.85	CCCCCCCCCCCCCCC1CO1
Oleic Acid	445639	C_18_H_34_O_2_	282.5	26.95	2140	12.56	CCCCCCCCC=CCCCCCCCC(=O)O
9,12-Octadecadienoic acid [Z]-, phenylmethyl ester	5368290	C_25_H_38_O	370.6	31.91	2766	11.14	CCCCCC=CCC=CCCCCCCCC(=O)OCC1=CC=CC=C1
5-Methyl-Z-5-docosene	5365995	C_23_H_46_	322.6	33.91	2292	3.94	CCCCCCCCCCCCCCCCC=C(C)CCCC
Ergost-5-en-3-ol, acetate, [3β,24R]-	13019955	C_30_H_50_O_2_	442.7	35.8	2771	10.46	CC(C)C(C)CCC(C)C1CCC2C1(CCC3C2CC=C4C3(CCC(C4)OC(=O)C)C)C
Stigmastan-6,22-dien, 3,5-dedihydro-	5364573	C_29_H_46_	394.7	36.56	2437	7.02	CCC(C=CC(C)C1CCC2C1(CCC3C2C=CC45C3(CCC4C5)C)C)C(C)C
6,9,12-Octadecatrienoic acid, phenylmethyl ester, [Z,Z,Z]-	5368209	C_25_H_36_O_2_	368.6	37.12	2774	1.09	CCCCCC=CCC=CCC=CCCCCC(=O)OCC1=CC=CC=C1
Tridecanedial	544162	C_13_H_24_O_2_	212.33	37.38	1690	2.02	C(CCCCCC=O)CCCCCC=O
9,10-Secocholesta-5,7,10[19]-triene-3,24,25-triol, [3β,5Z,7E]-	6434253	C_27_H_44_O_3_	416.6	37.54	3124	2.15	CC(CCC(C(C)(C)O)O)C1CCC2C1(CCCC2=CC=C3CC(CCC3=C)O)C
Stigmastan-3,5-diene	525918	C_29_H_48_	396.7	37.92	2525	15.93	CCC(CCC(C)C1CCC2C1(CCC3C2CC=C4C3(CCC=C4)C)C)C(C)C
6-Octadecenoic acid, methyl ester, [Z]-	5362717	C_19_H_36_O_2_	296.5	38.39	2085	1.95	CCCCCCCCCCCC=CCCCCC(=O)OC
9-Octadecenoic acid [Z]-, phenylmethyl ester	5368218	C_25_H_40_O_2_	372.6	39.1	2758	16.64	CCCCCCCCC=CCCCCCCCC(=O)OCC1=CC=CC=C1
Z-8-Methyl-9-tetradecenoic acid	5364410	C_15_H_28_O_2_	240.38	39.34	1813	10.25	CCCCC=CC(C)CCCCCCC(=O)O
E-11-Tetradecenol, trimethylsilyl ether	5366871	C_17_H_36_OSi	284.6	20.76	1705	1.02	CCC=CCCCCCCCCCCO[Si](C)(C)C

(iu: index unit).

**Table 3:** Functional groups characterization of *C. crassa* powder by FTIR analysis.

Frequency (cm^−1^)	Intensity	Assignments	Characterization
3648	Medium, Sharp	O-H stretching	alcohol
3566	Medium, Sharp	O-H stretching	alcohol
3346	Strong, broad	O-H stretching	alcohol
	Medium	N-H stretching	Aliphatic primary amine
2925	Strong, broad	O-H stretching	Carboxylic acid
2859	Strong, broad	O-H stretching	Carboxylic acid
	Weak, broad	O-H stretching	alcohol
	Strong broad	N-H stretching	Amine salt
	Medium	C-H stretching	Alkane
1888	Weak	C-H bending	Aromatic compound
1869	Weak	C-H bending	Aromatic compound
1714	Weak	C-H bending	Aromatic compound
	Strong	C=O stretching	Carboxylic acid
	Strong	C=O stretching	Aliphatic ketone
1660	Weak	C-H bending	Aromatic compound
	Medium	C=N stretching	Imine/oxime
	Medium	C=C stretching	Alkene
1540	Strong	N-O stretching	Nitro compound
1517	Strong	N-O stretching	Nitro compound
1432	medium	O-H Bending	Carboxylic acid
1382	Medium	O-H bending	alcohol
	medium	O-H bending	Phenol
1192	Medium	C-N stretching	Amine
	Strong	C-O stretching	Ester
	Strong	C-O stretching	Tertiary alcohol
1111	Medium	C-N stretching	Amine
	Strong	C-O stretching	Aliphatic ether
	Strong	C-O stretching	Primary alcohol
1041	Strong	S=O stretching	Sulfoxide
	Strong, broad	CO-O-CO Stretching	anhydride
875	Strong	C-H bending	1,2,4-trisubstituted
	Strong	C-H bending	1,3-disubstituted
674	Strong	C-Cl stretching	Halo compound
	Strong	C=C bending	alkene
	Strong	C-Br stretching	Halo compound

**Table 4:** Mineral contents of *C. crassa* by ICP-AES.

Minerals	Amount in %
B	ND
Ca	1.71
Cu	ND
Fe	0.86
K	6.91
Mg	0.58
Zn	0.02
Na	0.56
Si	26.28
Se	ND

ND means less than 0.01%.

**Table 5:** Determination of total amino acid profile of *C. crassa* by using HRLCMS-QTOF.

Sr.no	Amino Acids	g/kg
1	Aspartic acid	1.6
2	Glutamic Acid	1.3
3	Asparagine	ND
4	Serine	0.5
5	Glutamine	ND
6	Histidine	ND
7	Glycine	0.6
8	Threonine	0.2
9	Arginine	0.4
10	Alanine	0.7
11	Tyrosine	0.2
12	Cystine	ND
13	Valine	0.2
14	Methionine	0.2
15	Norvaline	ND
16	Tryptophan	ND
17	phenylalanine	0.4
18	Isoleucine	0.2
19	leucine	0.4
20	Lysine	0.4
21	Hydroxyproline	1.1

**Table 6:** Different carbohydrate derivatives and their chemical details obtained by HRLCMS-QTOF.

Name	PubChem ID	Molecular Formula	RT (min)	Mass (Da)	Hits (DB)
2-Methyl-2-butenyl beta-D-glucopyranoside	10753054	C_11_H_20_O_6_	5.099	248.1242	10
Antibiotic JI 20A	198272	C_19_H_39_N_5_O_9_	5.317	481.2736	9
N2′-Acetylgentamicin C1a	16069998	C_21_H_41_N_5_O_8_	7.11	491.2948	4
Loquatoside	156269	C_20_H_22_O_11_	7.813	438.1167	10
Feruloyl C1-glucuronide	102331585	C_16_H_18_O_10_	9.608	370.0897	10
N-butyl-1-deoxynojirimycin	23622616	C_10_H_21_NO_4_	1.296	219.1459	1
Bactobolin	54676871	C_14_H_20_Cl_2_N_2_O_6_	10.278	383.2	4

**Table 7:** Different phycocompounds and their chemical details obtained by HRLCMS-QTOF.

Name	PubChem ID	Molecular Formula	RT (min)	Mass	Hits (DB)
beta-Butoxyethyl nicotinate	14866	C_12_H_17_NO_3_	4.356	223.1197	7
Dihydrocapsaicin	107982	C_18_H_29_NO_3_	7.648	307.2128	4
Manumycin A	6438330	C_31_H_38_N_2_O_7_	9.103	550.261	1
16-methyl-6Z,9Z,12Zheptadecatrienoic acid	5312303	C_18_H_32_O_2_	12.749	278.2235	10
Axisothiocyanate 3	23425033	C_16_H_25_NS	1.465	263.1723	6
N-(1-Oxooctyl)glycine	84290	C_10_H_19_NO_3_	4.03	201.1374	7
Phytosphingosine	122121	C_18_H_39_NO_3_	10.01	317.2915	1
Tyr Val Phe	139658609	C_23_H_29_N_3_O_5_	3.896	427.2065	6
Tyr Lys Lys	145458707	C_21_H_35_N_5_O_5_	5.175	437.2657	9
Tiocarbazil	37523	C_16_H_25_NOS	1.112	279.167	5
Benzo[a]fluorene	9195	C_17_H_12_	2.217	216.0964	5
4-Hydroxy-3-nitrosobenzamide	443631	C_7_H_6_N_2_O_3_	2.871	166.0372	8
(S)-Indenestrol A	146460	C_18_H_18_O_2_	3.738	266.1346	10
Isocorydine (+)	10143	C_20_H_23_NO_4_	4.6	341.1614	10
3-α(S)-Strictosidine	161336	C_27_H_34_N_2_O_9_	5.642	530.2246	10
Metaxalone	15459	C_12_H_15_NO_3_	5.243	221.1042	10
Scutellarioside II	6443034	C_24_H_28_O_12_	6.469	508.1563	9
Triphenylphosphine oxide	13097	C_18_H_15_OP	10.101	278.085	10
Pirimicarb	31645	C_11_H_18_N_4_O_2_	26.718	238.14	4
3,5-Dichloro-4-hydroxy-2methoxy-6-methylbenzoic acid	21724963	C_9_H_8_Cl_2_O_4_	28.406	249.9783	2
Dicyclohexyl disulfide	17356	C_12_H_22_S_2_	26.661	230.1166	1
Tetradecylamine	16217	C_14_H_31_N	10.446	213.2451	1
34a-Deoxy-rifamycin W	11017668	C_35_H_45_NO_10_	9.61	639.3083	4
(+/−)-3-[(2-methyl-3furyl)thio]-2-butanone	12980878	C_9_H_12_O_2_S	0.784	184.0575	3
Chinomethionat	17109	C_10_H_6_N_2_OS_2_	0.849	233.9916	3
Gabapentin	3446	C_9_H_17_NO_2_	1.063	171.1254	1
Azoxy-1-procarbazine	48599	C_12_H_17_N_3_O_2_	1.189	235.1349	4
2,2,6,6-Tetramethyl-4piperidinone	13220	C_9_H_17_NO	1.246	155.1305	4
beta-Butoxyethyl nicotinate	14866	C_12_H_17_NO_3_	2.902	223.1197	7

## Supplementary Materials

The following are available online, Table S1: Chemical structures obtained in Methanolic extract of *C. crassa*. Table S2: Details of Phycochemicals identified through GC-MS Analysis of Methanolic Extract. Table S3: Chemical structures of carbohydrate derivatives obtained by HRLCMS-QTOF in *C. crassa*. Table S4: Chemical structures obtained by HRLCMS-QTOF in *C. crassa*.

## Sample Availability

Samples of the compounds are available from the authors.
