# Supplementary material for: Characterization of Fatty Acids, Polysaccharides, Amino Acids, and Minerals in Marine Macroalga Chaetomorpha crassa and Evaluation of Their Potentials in Skin Cosmetics
Source: Molecules. 2021 Dec 11;26(24):7515. doi: 10.3390/molecules26247515 (PMC8706032; doi:10.3390/molecules26247515)
Supplement: Supplementary file 1 [file molecules-26-07515-s001.zip › molecules-1454126-si.pdf]

Table S1. Chemical structures obtained in Methanolic extract of *C. crassa*.

No.	Name of the Chemical Compound	Structures	Kovats Index (iu) in Present Study	Active Phase	Kovats Index Value from the Previous Study	Reference
1.	Tritriacontane, 13-decyl-13-heptyl-	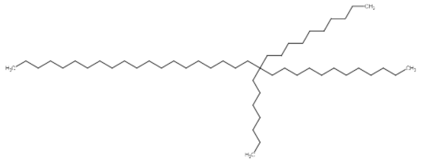	4907	DB-5MS	500.99	[1]
2.	Hexadecane	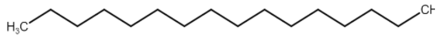	1612	DB-5MS<br>DB-5MS	268.29<br>272.02	[2]<br>[2]
3.	Hydroperoxide, 1-methylhexyl	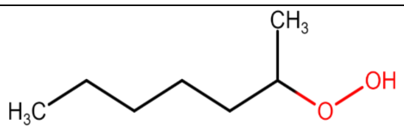	1013	-	-	-
4.	Oxalic acid, isobutyl nonyl ester	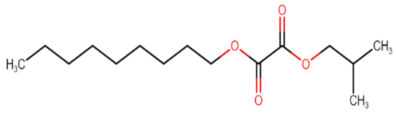	1783	-	-	-
5.	Dodecanoic acid,1,2,3-propanetriyl ester	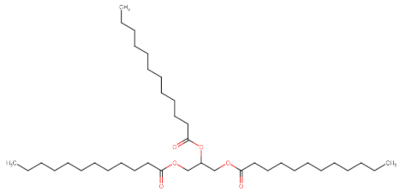	4336	-	-	-
6.	Phthalic acid, 6-ethyl-3-octyl butyl ester	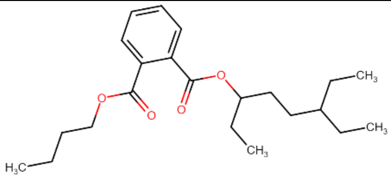	2505	5% Phenyl methyl siloxane	2209	[3]

(iu: index unit).

Table S2. Details of Phycochemicals identified through GC-MS Analysis of Methanolic Extract.

No.	Name of the Chemical Compound	Structures	Kovats Index (iu) in Present Study	Active Phase	Kovats Index Value from the Previous Study	Reference
1.	n-Hexadecanoic acid	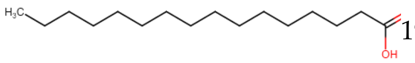	1968	BP-1<br>HP-5MS<br>DB-5<br>SE-30<br>HP-5<br>RTX-1	1961.<br>1964<br>1972<br>1940<br>1970<br>1942	[4]<br>[5]<br>[6]<br>[7]<br>[8]<br>[9]
2.			1702	HP-5 MS	1708	[10]

	Oxirane, tetradecyl-	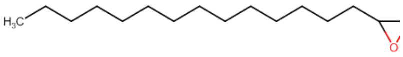		TR-5 MS	1676	[11]
3.	Oleic Acid	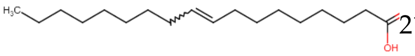	2140	HP-5<br>HP-5MS<br>DB-5<br>DB-Wax	2144<br>2141<br>2161<br>3184	[12]<br>[13]<br>[14]<br>[15]
4.	9,12-Octadecadienoic acid [Z]-, phenylmethyl ester	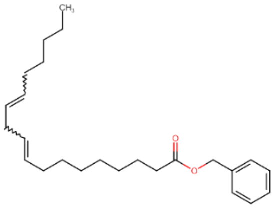	2766	HP-5MS	2764.5	[16]
5.	5-Methyl-Z-5-docosene	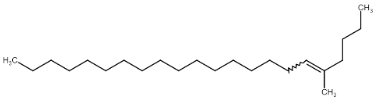	2292	-	-	-
6.	Ergost-5-en-3-ol, acetate, [3β,24R]-	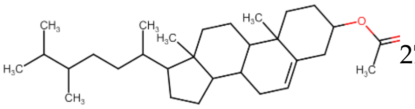	2771	OV-1<br>OV-1	3323<br>3278	[17]<br>[17]
7.	Stigmastan-6,22-dien, 3,5-dedihydro-	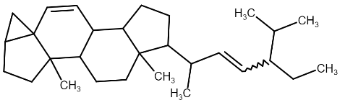	2437	-	-	-
8.	6,9,12-Octadecatrienoic acid, phenylmethyl ester, [Z,Z,Z]-	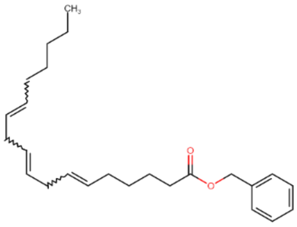	2774	HP-5	2702	[18]
9.	Tridecanedial	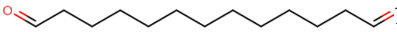	1690	HP-5	2229	[19]
10.	9,10-Secocholesta-5,7,10[19]-triene-3,24,25-triol, [3β,5Z,7E]-	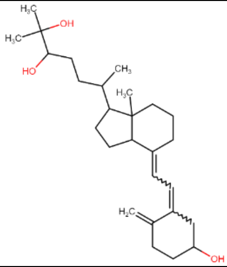	3124	-	-	-
11.	Stigmastan-3,5-diene	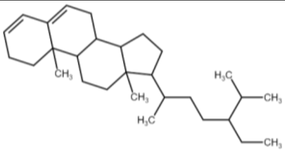	2525	OV-101	3040	[20]
12.	6-Octadecenoic acid, methyl ester, [Z]-	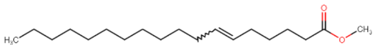	2085	VF-5MS	2104.5	[21]
13.	9-Octadecenoic acid [Z]-,		2758	OV-101<br>CP Sil 5 CB<br>DB-1	2074.7<br>2072<br>2085	[22]<br>[23]<br>[24]

	phenylmethyl ester	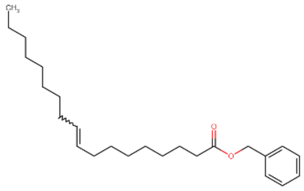		SE-30<br>DB-5	2081<br>2085	[25]<br>[26]
14.	Z-8-Methyl-9-tetradecenoic acid	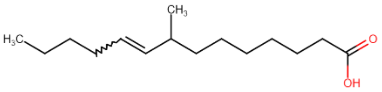	1813	-	-	-
15.	E-11-Tetradecenol, trimethylsilyl ether	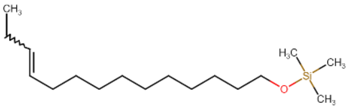	1705	-	-	-

(iu: index unit).

Table S3. Chemical structures of carbohydrate derivatives obtained by HRLCMS-QTOF in *C. crassa*.

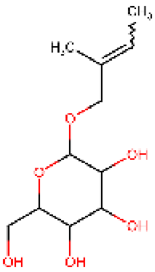	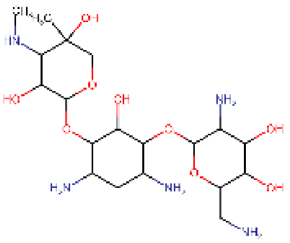	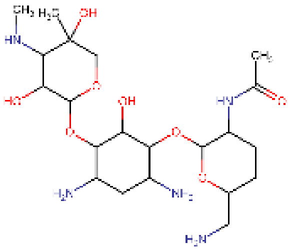	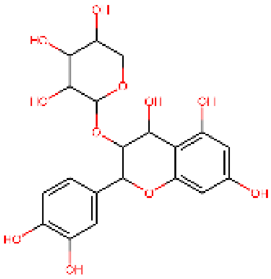
10753054	198272	16069998	156269
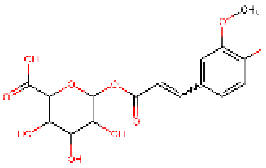	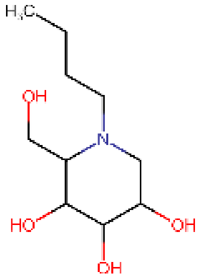	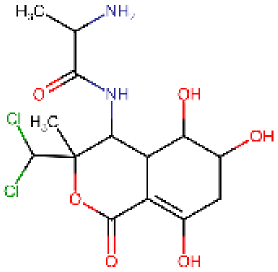	
102331585	23622616	54676871	

(Each obtained chemical structure labeled with its PubChem ID; PubChem ID tabulated in Table 6).

Table S4. Chemical structures obtained by HRLCMS-QTOF in *C. crassa*.

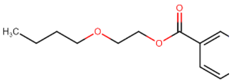	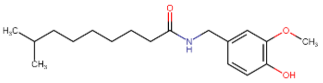	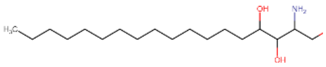	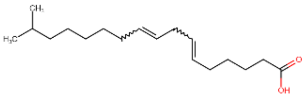
14866	107982	122121	5312303

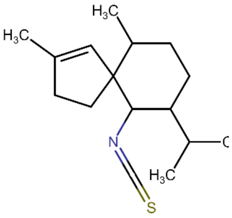

23425033

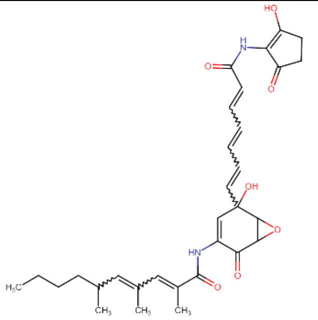

6438330

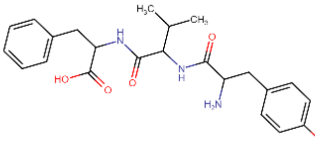

139658609

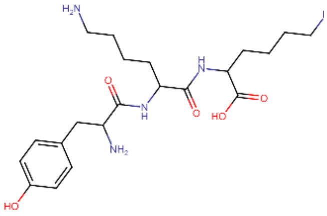

145458707

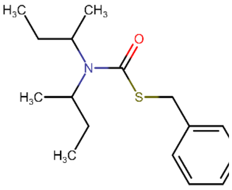

37523

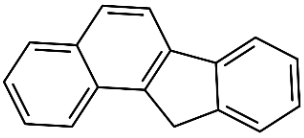

9195

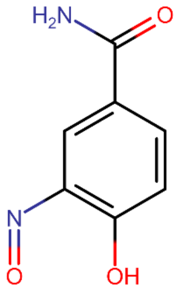

443631

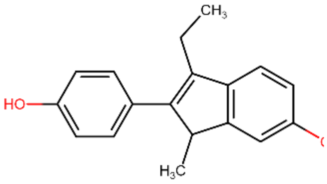

146460

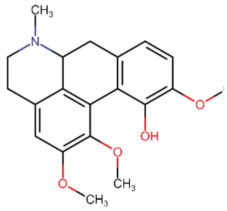

10143

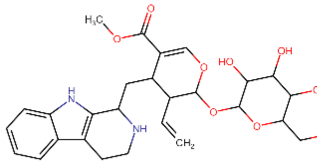

161336

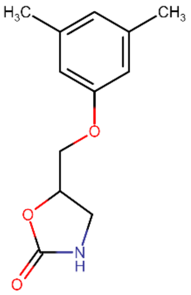

15459

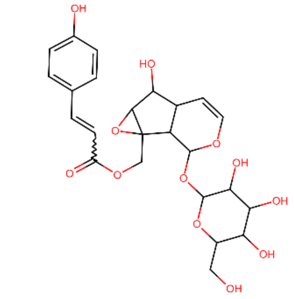

6443034

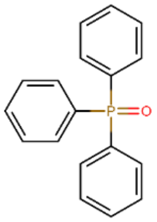

13097

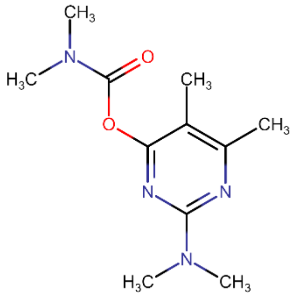

31645

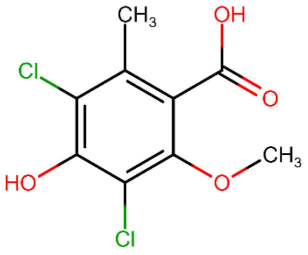

21724963

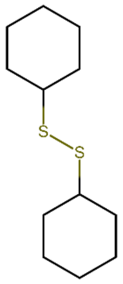

17356

16217

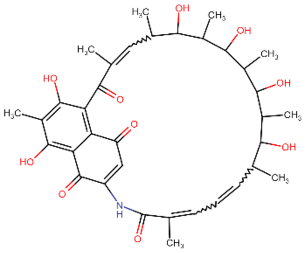

11017668

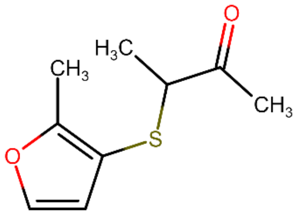

12980878

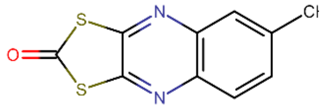

17109

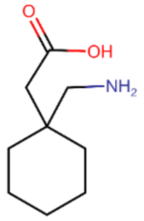

3446

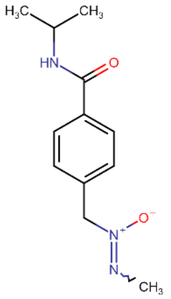

48599

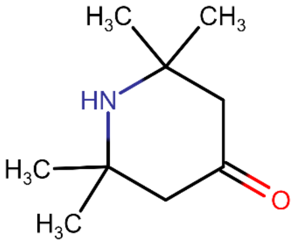

13220

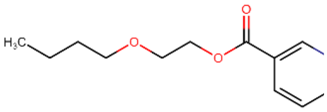

14866

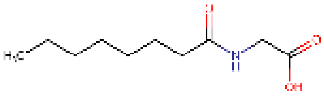

84290

(Each obtained chemical structure labeled with its PubChem ID; PubChem ID tabulated in Table 7).

Reference

1. Chen, P.H.; Keeran, W.S.; Van Ausdale, W.A.; Schindler, D.R.; Roberts, D.W. Application of Lee retention indices to the confirmation of tentatively identified compounds from GC/MS analysis of environmental samples. In Proceedings of the Ninth Annual Waste Testing and Quality Assurance Symposium, Washington, DC, USA, July 12-16, **1993**.
2. Chen, P.H.; Keeran, W.S.; Van-Ausdale, W.A.; Schindler, D.R.; Roberts, D.W. *Technical paper, Analytical Services Division*; Environmental Science and Engineering, Inc., PO Box 1703, Gainesville, FL 32602, 11**(2002)**.
3. Zaikin, V.G. Chromatography-mass spectrometry. *J. Anal. Chem.* **2011**, *66*, 1090–1094.
4. Raina, V.K.; Verma, S.C.; Dhawan, S.; Khan, M.; Ramesh, S.; Singh, S.C.; Yadav, A.; Srivastava, S.K. Essential oil composition ofMurraya exotica from the plains of northern India. *Flavour Fragr. J.* **2006**, *21*, 140–142. https://doi.org/10.1002/ffj.1547.
5. Asuming, W.A.; Beauchamp, P.S.; Descalzo, J.T.; Dev, B.C.; Dev, V.; Frost, S.; Ma, C.W. Essential oil composition of four Lomatium Raf. species and their chemotaxonomy. *Biochem. Syst. Ecol.* **2005**, *33*, 17–26. https://doi.org/10.1016/j.bse.2004.06.005.
6. Morteza-Semnani, K.; Azadbakht, M.; Goodarzi, A. The essential oils composition of Phlomis herba-venti L. leaves and flowers of Iranian origin. *Flavour Fragr. J.* **2004**, *19*, 29–31.
7. Jantan, I.; Ahmad, A.S.; Ahmad, A.R.; Ali, N.A.M.; Ayop, N. Chemical Composition of Some Citrus Oils from Malaysia. *J. Essent. Oil Res.* **1996**, *8*, 627–632. https://doi.org/10.1080/10412905.1996.9701030.
8. Stojanovic, G.; Palic, R.; Alagic, S.; Zeković, Z. Chemical composition and antimicrobial activity of the essential oil and CO2 extracts of semi-oriental tobacco, Otlja. *Flavour Fragr. J.* **2000**, *15*, 335–338. https://doi.org/10.1002/1099-1026(200009/10)15:53.3.co;2-n.
9. Paolini, J.; Muselli, A.; Bernardini, A.-F.; Bighelli, A.; Casanova, J.; Costa, J. Thymol derivatives from essential oil ofDoronicum corsicum L. *Flavour Fragr. J.* **2007**, *22*, 479–487. https://doi.org/10.1002/ffj.1824.
10. Xu, L.-L.; Han, T.; Wu, J.-Z.; Zhang, Q.-Y.; Zhang, H.; Huang, B.-K.; Rahman, K.; Qin, L.-P. Comparative research of chemical constituents, antifungal and antitumor properties of ether extracts of Panax ginseng and its endophytic fungus. *Phytomedicine* **2009**, *16*, 609–616. https://doi.org/10.1016/j.phymed.2009.03.014.
11. Kurashov, E.A.; Mitrukova, G.G.; Krylova, Y.V. Dynamics of essential oil composition in Ceratophyllum demersum (Ceratophyllaceae) during vegetation. *Rastit. Resur.* **2014**, *50*, 132–144.
12. Leffingwell, J.C.; Alford, E.D. Volatile constituents of perique tobacco. *Electron. J. Environ. Agric. Food Chem.* **2005**, *4*, 899–915.
13. Pino, J.A.; Mesa, J.; Muñoz, Y.; Martí, M.P.; Marbot, R. Volatile Components from Mango (*Mangifera indica* L.) Cultivars. *J. Agric. Food Chem.* **2005**, *53*, 2213–2223. https://doi.org/10.1021/jf0402633.
14. A Priestap, H.; van Baren, C.M.; Lira, P.D.L.; Coussio, J.; Bandoni, A.L. Volatile constituents of Aristolochia argentina. *Phytochemistry* **2003**, *63*, 221–225. https://doi.org/10.1016/s0031-9422(02)00751-3.
15. Chung, T.Y.; Eiserich, J.P.; Shibamoto, T. Volatile compounds produced from peanut oil heated with different amounts of cysteine. *J. Agric. Food Chem.* **1994**, *42*, 1743–1746. https://doi.org/10.1021/jf00044a032.
16. Andriamaharavo, N.R. 2014, Retention data. NIST Mass Spectrometry Data Center. Available online: https://webbook.nist.gov/cgi/inchi?ID=C60045274&Mask=2000 (accessed on November 16, 2021).
17. Itoh, T.; Tani, H.; Fukushima, K.; Tamura, T.; Matsumoto, T. Structure-retention relationship of sterols and triterpene alcohols in gas chromatography on a glass capillary column. *J. Chromatogr. A* **1982**, *234*, 65–76. https://doi.org/10.1016/s0021-9673(00)81781-1.

18. Zhao, Y.-P.; Wang, X.-Y.; Wang, Z.-C.; Lu, Y.; Fu, C.-X.; Chen, S.-Y. Essential oil of Actinidia macrosperma, a catnip response kiwi endemic to China. *J. Zhejiang Univ. Sci. B* **2006**, *7*, 708–712. https://doi.org/10.1631/jzus.2006.b0708.
19. Zeng, Q.Y.; Wu, J.; Lin, P.C. Chemical Composition and Antimicrobial Activity of the Essential Oil from Epilobium angustifolium. *Chem. Nat. Compd.* **2016**, *52*, 1113–1115. https://doi.org/10.1007/s10600-016-1878-y.
20. Velisek, J.; Davidek, J.; Kubelka, V. Formation of DELTA.3,5-diene and 3-chloro DELTA.5-ene analogs of sterols in protein hydrolysates. *J. Agric. Food Chem.* **1986**, *34*, 660–662. https://doi.org/10.1021/jf00070a017.
21. Tret'yakov, K.V. Retention Data NIST Mass Spectrometry Data Center. 2007. Available online: http: l/webbook: nist. govlcgilcbook.cgi (accessed on November 18, 2021).
22. Kittiratanapiboon, K.; Jeyashoke, N.; Krisnangkura, K. The Relationship of Kovάts Retention Indices and Equivalent Chain Lengths of Fatty Acid Methyl Esters on a Methyl Silicone Capillary Column. *J. Chromatogr. Sci.* **1998**, *36*, 361–364.
23. Hanai, T.; Hong, C. Structure-retention correlation in CGC. *J. High Resolut. Chromatogr.* **1989**, *12*, 327–332. https://doi.org/10.1002/jhrc.1240120517.
24. Golovnya, R.V.; Kuzmenko, T.E. Thermodynamic evaluation of the interaction of fatty acid methyl esters with polar and non-polar stationary phases, based on their retention indices. *Chromatographia* **1977**, *10*, 545–548. https://doi.org/10.1007/bf02262915.
25. Golovnya, R.; Uralets, V.; Kuzmenko, T. Characterization of fatty acid methyl esters by gas chromatography on siloxane liquid phases. *J. Chromatogr. A* **1976**, *121*, 118–121. https://doi.org/10.1016/s0021-9673(00)82312-2.
26. Wu, S.; Zorn, H.; Krings, U.; Berger, R.G. Volatiles from submerged and surface-cultured beefsteak fungus,Fistulina hepatica. *Flavour Fragr. J.* **2007**, *22*, 53–60. https://doi.org/10.1002/ffj.1758.
